# Tolerance to environmental pollution in the freshwater crustacean *Asellus aquaticus*: A role for the microbiome

**DOI:** 10.1111/1758-2229.13252

**Published:** 2024-05-23

**Authors:** Terézia Horváthová, Elvira Lafuente, Jo‐Anne Bartels, Jesper Wallisch, Christoph Vorburger

**Affiliations:** ^1^ Department of Aquatic Ecology Eawag Dübendorf Switzerland; ^2^ Institute of Soil Biology and Biochemistry Biology Centre CAS České Budějovice Czechia; ^3^ Instituto Gulbenkian de Ciência Oeiras Portugal; ^4^ D‐USYS, Department of Environmental Systems Science ETH Zürich Zürich Switzerland

## Abstract

Freshwater habitats are frequently contaminated by diverse chemicals of anthropogenic origin, collectively referred to as micropollutants, that can have detrimental effects on aquatic life. The animals' tolerance to micropollutants may be mediated by their microbiome. If polluted aquatic environments select for contaminant‐degrading microbes, the acquisition of such microbes by the host may increase its tolerance to pollution. Here we tested for the potential effects of the host microbiome on the growth and survival of juvenile *Asellus aquaticus*, a widespread freshwater crustacean. Using faecal microbiome transplants, we provided newly hatched juveniles with the microbiome isolated from donor adults reared in either clean or micropollutant‐contaminated water and, after transplantation, recipient juveniles were reared in water with and without micropollutants. The experiment revealed a significant negative effect of the micropollutants on the survival of juvenile isopods regardless of the received faecal microbiome. The micropollutants had altered the composition of the bacterial component of the donors' microbiome, which in turn influenced the microbiome of juvenile recipients. Hence, we show that relatively high environmental concentrations of micropollutants reduce survival and alter the microbiome composition of juvenile *A. aquaticus*, but we have no evidence that tolerance to micropollutants is modulated by their microbiome.

## Graphical abstract

Testing the effects of micropollutants on the growth, survival and microbiome of juvenile isopods *Asellus aquaticus* using the faecal microbiome transplants.

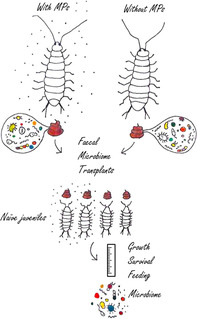

## INTRODUCTION

Environments are changing dramatically under human influence. To cope with these changes, organisms have to adjust their development, physiology or behaviour to improve their chances of surviving and reproducing (e.g., Huey et al., 2012; Tuomainen & Candolin, 2011). The selection imposed by changing environmental conditions can eventually lead to genetic adaptation, but in the face of rapid change, organisms can also respond without the need for changes in genetic composition (e.g., via phenotypic plasticity; Nussey et al., 2007). Host‐associated microbiomes fulfil important ecological functions that influence host fitness (McFall‐Ngai et al., 2013), and they can show rapid compositional modification in response to environmental change (Martiny et al., 2023; Ziegler et al., 2019), making them a potential mechanism for rapid acclimation and adaptation of individuals to a changing environment (Voolstra & Ziegler, 2020). These environment‐driven changes in microbiome composition have been hypothesised to provide a special form of plasticity (Henry et al., 2021; Kolodny & Schulenburg, 2020).

The microbiome has emerged as a key determinant of many aspects of organismal biology, shaping host immunity, behaviour, metabolism, and reproduction (Bang et al., 2018; Gilbert et al., 2012). It is composed of bacteria, fungi, protozoa, archaea, and viruses that live in and on the host body, with a majority residing in the digestive tract (e.g., >90% in humans, Sender et al., 2016), and with no distinction between microbes with beneficial, neutral, or detrimental effects on their hosts (Drew et al., 2021; Engel & Moran, 2013). Gut microbiomes have been found to vary temporally (Holt et al., 2020; Koliada et al., 2020), within as well as between individuals and populations (Tavalire et al., 2021; Vandeputte et al., 2021) and they can be strongly shaped by various environmental factors such as diet (Youngblut et al., 2019; Yun et al., 2014), temperature (Huus & Ley, 2021; Li et al., 2023; Sepulveda & Moeller, 2020), exposure to pathogens (González & Elena, 2021) or anthropogenic impacts (Lavrinienko et al., 2018; Zhu et al., 2022). Not only the host's environment, but also the host genotype can contribute to shaping the gut microbiome (Bulteel et al., 2021; Qin et al., 2022), though microbiome heritability is generally considered to be low to moderate (Grieneisen et al., 2021; Ryu & Davenport, 2022). Ultimately, it is the combination of host genetics and environment that contribute to the observed microbiome variation (Callens et al., 2020; Griffiths et al., 2019). The microbiome is increasingly documented as one of the drivers of host adaptive changes (Bruijning et al., 2022; Henry et al., 2021), with evidence for microbiome‐mediated local adaptation to thermal stress (Baldassarre et al., 2022), exposure to toxins (Kikuchi et al., 2012), aridity stress (Ribeiro et al., 2019), pollution (Claus et al., 2016) or dietary challenge (Bisschop et al., 2022; Wagener et al., 2020). The mechanisms behind the increased tolerance to stressors provided by the microbiome are often not fully understood, since microbiomes are usually very complex and comprise multiple microbes with diverse functional (metabolic) versatility to assist the host in adaptation to changing conditions.

Fresh waters and freshwater organisms are particularly vulnerable to changing environmental conditions, especially to increased ambient temperature and elevated atmospheric CO_2_ (Woodward et al., 2010) as well as to pollution and habitat fragmentation (Reid et al., 2019). Consequently, freshwater ecosystems have suffered more than 80% decline of monitored species over the last 50 years (WWF, 2022). Aquatic animals are naturally surrounded by a milieu of microbes, which perform numerous biological functions, but also serve as a source of microbes for the host microbiome (Sehnal et al., 2021). Evidence suggests that the composition of aquatic microbiota strongly influences the microbiomes of the hosts. In filter‐feeding zooplankton, the majority of microbes is acquired from the surrounding water (Callens et al., 2020; Eckert et al., 2020). In larger freshwater vertebrates, the gut microbiome is not simply a reflection of the surrounding milieu but is rather shaped by host trophic level, habitat (e.g., salinity) and host phylogeny (Kim et al., 2021; Sullam et al., 2012). However, in comparison to terrestrial animal microbiomes, the gut microbiome of aquatic animals is more flexible and highly sensitive to environmental perturbations (e.g., Sehnal et al., 2021). Therefore, continuous recruitment of microbes from the surrounding environment by freshwater organisms might provide one of the mechanisms of coping with environmental changes. More specifically, microbiome‐mediated tolerance to environmental pollution could be linked to the capacity of specific microbes to metabolise environmental chemicals, thus modulating the toxicity for the host (Claus et al., 2016; Itoh et al., 2018).

In this study, we examined the potential role of the microbiome in coping with environmental pollution in a freshwater isopod. *Asellus aquaticus* (Linnaeus 1758) (Asellota: Asellidae) is one of the most common freshwater detritivorous crustaceans widely distributed throughout Europe (Lafuente et al., 2023; Verovnik & Konec, 2019). As an extreme generalist, *A. aquaticus* can survive and reproduce in a wide range of ecologically different habitats and on different food sources (Lafuente et al., 2021; Verovnik et al., 2005). It is a keystone species, fulfilling important ecosystem services by decomposing plant and animal material (Fleeger et al., 2003). The species tolerates a wide range of environmental pollutants including polycyclic aromatic hydrocarbons, endocrine‐disrupting compounds, radionuclide contaminants or nanoparticles (Maltby, 1991; O'Callaghan et al., 2019), and is able to accumulate heavy metals (Goodyear & McNeill, 1999). The gut microbiome is typically diverse and differs both at the individual and at the population level (Lafuente et al., 2023; Liao et al., 2023), with a considerable fraction of the gut microbes derived from environmental sources (Liao et al., 2023). We aimed to assess whether the bacterial component of the microbiome of *A. aquaticus* plays a role in coping with environmental pollution by so‐called micropollutants (MPs), including pesticides, pharmaceuticals, consumer products, and industrial chemicals, using faecal microbial transplants and fitness quantification of naïve juveniles. In our study, we (1) investigated the changes in host‐associated microbiomes induced by the presence of MPs in water, and (2) tested whether transplanting the faecal microbiome from individuals previously exposed to MPs to naïve juveniles improves their growth, survival and/or feeding rates under stressful conditions (i.e., exposure to MPs).

## EXPERIMENTAL PROCEDURES

### Experimental animals

Individuals of *A. aquaticus* were collected with sampling nets from the lakebed of an unpolluted nearshore area (littoral zone) of lake Lucerne, Switzerland (47.001940, 8.333329) during the spring breeding season (June 2021). The animals were placed in boxes filled with lake water and some leaves as food and transported immediately to the laboratory. Until the start of the experiment, animals were maintained in a flow‐through system in 4.5‐L tanks, at a density of ~100 animals of both sexes per tank with a temperature of 18°C and a light:dark cycle of 16:8 h. The animals were reared in animal proof water (tap water with minimal concentrations of heavy metals) and fed with pre‐soaked alder (*Alnus glutinosa*) leaves.

### Experimental design

A schematic representation of the experimental design is depicted in Figure S1. The microbiome donor populations were established by placing 15 adult male and 15 adult female field‐collected *A. aquaticus* into each of eight 1.5 L tanks for a period of 2 weeks with ad libitum alder leaves. In four of the tanks, donor individuals were exposed to MPs (treatment MP+) and in the other four tanks to normal animal proof water without any MPs (treatment MP−), resulting in a total of 120 donor individuals per donor treatment. The MPs comprised 17 organic compounds including 10 pharmaceuticals, four pesticides, one sweetener, one hormone, and one corrosion inhibitor (Table 1). They were provided in the form of stock solutions provided by the EcoImpact consortium at Eawag, the Swiss Federal Institute of Aquatic Science and Technology (Table 1). The composition and concentrations of the mixture are based on the measured levels of MPs that typically occur in waste‐water impacted Swiss rivers and streams (Stamm et al., 2016). Depending on the solubility of each compound, MPs were dissolved either in methanol or in Nanopure water (see Table 1). We used twice the highest average concentration of MPs found in Swiss streams near wastewater effluents (see Table 1; Stamm et al., 2016). Water (with or without MPs) was exchanged once a week. After the two‐week incubation period in either of the treatments, donor individuals were placed in new tanks and starved for 6 h to collect freshly produced faecal pellets for the microbiome transplants. The faeces were isolated from the tanks and pooled together within MP+ and MP‐ treatments, thus generating F+ (polluted) and F− (unpolluted) faecal microbiome (donor) transplants (see Figure S1). After 2 weeks in the incubation treatment, the total number of surviving donors was 82 (in MP+) and 89 (in MP−), a difference that was not statistically significant (Fisher's exact test, *p* = 0.392).

**TABLE 1:** The composition and concentrations of MPs used in the experiment. Two concentrations of a MP mixture (MP_LOW, MP_HIGH) were previously used to experimentally test responses of aquatic organisms to levels of MPs measured in Swiss rivers and streams (Taddei et al. 2021; Salo et al. 2017) based on Stamm et al. (2016). Our micropollutant concentrations were based on two times high concentration (MP_HIGH) to observe detectable effects of MPs on performance of freshwater isopod *Asellus aquaticus*.

			MPs concentration used in previous experiments		MPs concentration used in the experiment
MP	Category	Solvent	MP_LOW (ng/L)	MP_HIGH (ng/L)	MP_2HIGH (ng/L)
Amisulpride	Pharmaceutical	MeOH	106.01	212.01	424.02
Atenolol	Pharmaceutical	MeOH	215.87	431.74	863.49
Benzotriazole	Corrosion inhibitor	MeOH	1093.80	2187.59	4375.19
Carbamazepine	Pharmaceutical	MeOH	282.44	564.88	1129.76
Citalopram HBr	Pharmaceutical	MeOH	55.21	110.41	220.83
Clarithromycin	Pharmaceutical	MeOH	62.90	125.81	251.61
Diazinon	Insecticide	MeOH	451.90	903.80	1807.60
Diclofenac	Pharmaceutical	MeOH	599.65	1199.30	2398.61
Diuron	Herbicide	MeOH	74.55	149.11	298.22
Estradiol	Estrogenic hormone	MeOH	0.35	0.69	1.38
Fexofenadine HCl	Pharmaceutical	MeOH	320.05	640.11	1280.22
Tebuconazole	Fungicide	MeOH	24.09	48.18	96.36
Triclosan	Biocide	MeOH	55.53	111.05	222.11
Valsartan	Pharmaceutical	MeOH	675.21	1350.41	2700.83
Iopromide	Pharmaceutical	H_2_O	1624.00	3248.00	6496.01
Sucralose	Artificial sweetener	H_2_O	1170.21	2340.42	4680.83
Metformin HCl	Pharmaceutical	H_2_O	5035.37	10070.73	20141.46

*Note*: **Amisulpride,** member of the class of benzamides, is an antipsychotic drug; **Atenolol** is an ethanolamine compound used as cardio‐selective beta‐blocker; **Benzotriazole** is a heterocyclic compound used in cosmetics, household products and corrosion inhibitors; **Carbamazepine** is a tricyclic drug used against epilepsy; **Citalopram Hydrobromide** is an active ingredient in antidepressants; **Clarithromycin** is a macrolide antibiotic, inhibiting RNA‐dependent protein synthesis in the target organisms; **Diazinon** is an organophosphorus pesticide; **Diclofenac** is a nonsteroidal benzene acetic acid used to treat the body pain, inflammations and fever; **Diuron** is a photosynthesis inhibitor used as herbicide for general weed control; **Estradiol** is used therapeutically as a synthetic form of the steroid sex hormone estradiol in hormone replacement therapy; **Fexofenadine Hydrochloride** is an antihistamine; **Tebuconazole** is a triazole fungicide used in a plant protection; **Triclosan** is widely used as preservative and antimicrobial agent in personal care and household products; **Valsartan** is an angiotensin II receptor blocker to treat hypertension; **Iopromide** is administrated as iodinated contrast medium for X‐ray imaging; **Sucralose** is an artificial sweetener and **Metformin Hydrochloride** is a drug used to treat high blood sugar in diabetic patients.

The recipient symbiont‐naïve population was established by placing individual gravid females in wells of 3 × 2 well plates filled with water and an alder leaf disc. The pregnancy in isopods can be easily determined by the presence of a brood pouch (Maltby, 1991). Brood pouch development took approximately 2 weeks and 16 broods amounting to 300 juveniles hatched within the same 3 days were used for the faecal microbiome transplant experiment. We set up a two‐factorial design to test the effect of faecal microbiome transplant on juvenile isopod performance (Figure S1). Recipient juveniles were reared either in polluted or unpolluted water (treatments W+ and W−, respectively), and supplemented with the faecal transplant from the polluted or unpolluted donor population (treatments F+ and F−, respectively) resulting in four experimental treatments: W + F+, W + F−, W−F+, W−F−. Juveniles from the same brood were split and distributed equally among the four treatments (Figure S1). Juveniles were reared in single wells (6 × 2 well plates) filled with 800 μL of MP+ or MP− water and were provided with a size‐standardised (90 mm^2^) alder leaf disc. We used the same MPs stock solution as for the donor population. Single faecal pellets originating from MP+ or MP− donors were provided to juvenile recipients only once at the beginning of the experiment. After that, juvenile survival was checked every 48–72 h (see the next section). In the well plates, water changes were performed once per week by pipetting the water out and replacing it with fresh water of the corresponding treatment. The experiment was terminated when only 10 individuals remained alive in one of our experimental treatments, which was 21 days after the start of the experiment.

### Quantification of juvenile performance

Based on the assumption that MPs can have a negative effect on isopod performance (Lafuente et al., 2023; Maltby, 1991), we quantified three host traits: survival, growth (i.e., body length increase), and food consumption. Survival was assessed by visual inspection of the juveniles every 48–72 h over a period of 21 days. Growth and food consumption were quantified from pictures taken at the beginning and the end of the experiment under constant light conditions with a Canon EOS 750D camera mounted on a repro stand. Growth was measured as the difference between the initial and final body sizes, which was calculated as the length from the tip of the cephalon to the end of the telson (excluding antennae and uropods). Food consumption was measured as the difference between the surface area of the leaf disc at the beginning and the end of an experiment. Pictures were analysed using FiJi ImageJ2 (Schindelin et al., 2012).

### DNA extraction and 16S library preparation

Four different types of samples were taken to document potential changes in the host microbiome in response to the experimental treatments: (1) donor (adult) individuals were used to analyse the effect of MPs on their microbiome (*N* = 4 adults per tank, *N* = 16 per MP treatment); (2) faecal samples, collected from the donor tanks after 2 weeks, were used to identify if MPs induced changes in the faecal microbiome (*N* = 3 faecal pellets per MP treatment); (3) a subset of recipient juveniles sacrificed 48 h after the faecal transplant (“early recipient juveniles”) was used to determine if the microbiome transplant had influenced the juvenile microbiome (*N* = 4 juveniles originating from the same brood per treatment); (4) recipient juveniles collected at the end of the experiment (“recipient juveniles”) were analysed to assess potential effects of rearing treatment and faecal transplant by the end of the experiment (*N* = 10 juveniles per treatment). Individuals were surfaced‐sterilised with a bleach wash followed by two consecutive washes with Milli‐Q water. Faecal pellets or whole bodies were placed in individual Eppendorf tubes and immediately stored at −80°C until DNA extraction (see detailed sample list in Table S1).

DNA was extracted from the samples using Qiagen DNeasy Blood & Tissue Kit (QIAGEN N.V, 2006) according to the manufacturer's instructions and immediately frozen at −20°C. The final number of samples used for 16S rRNA gene amplicon sequencing was 94 (see Table S1), including 32 donor samples, six donor faeces samples, 16 early recipient juvenile samples, and 40 recipient juvenile samples. DNA negative controls included two extraction controls and two PCR negative controls. The assessment of DNA quality and quantity was carried out using NanoDrop and Qubit 2.0 (Invitrogen™). The bacterial 16S rRNA gene's variable region V3‐V4 was amplified with universal bacterial primers b341F (5′‐CCTACGGGAGGCAGCAG‐3′) and 785R (5′‐CTACCAGGGTATCTAATCC‐3′), which were adapted for library preparation and Illumina MiSeq sequencing by adding Nextera adapters, 0–3 bp random frameshifts, and a 19‐bp Multiplex Identifier sequence (Table S1). The PCR reactions (25 μL) were performed in triplicates and pooled for each sample. Each PCR reaction contained 1× Qiagen Multiplex PCR MasterMix, 0.3 μM of both forward and reverse primer, and 3 μL s of a template DNA. The PCR cycles were performed as follows: 95°C for 15 min, followed by 32 cycles for juveniles (early recipient and recipient juveniles) due to low DNA concentrations and 31 cycles for the other samples (donor faeces and donor adults), of 95°C for 45 s, 55°C for 60 s, 72°C for 60 s and a final extension of 72°C for 10 min. PCR products were purified twice with AMPure XP beads from Beckman CoulterTM. Indexed PCR products were amplified for 10 cycles with Nextera XT v2 indexing primers using KAPA HiFi HotStart ReadyMix (Roche Holding AG, Basel, Switzerland), followed by another two‐step purification with AMPure XP beads. The concentration of each sample library was determined by Qubit (dsDNA‐Assay, Spark 10 M device) prior to normalisation. In total, 96 separate libraries were prepared including four reagent‐only negative controls from the DNA extraction step pooled together as well as two reagent‐only controls from the PCR step. The final library pool was quantified using Qubit (dsDNA‐Assay, Spark 10 M device) and Tape Station (Agilent Technologies, Santa Clara, USA). Pooled libraries were sequenced on the Illumina MiSeq (Illumina, San Diego, USA) using a 600 cycle v3 sequencing kit, paired‐end 300 cycle sequencing modes at the Genetic Diversity Center (GDC) of ETH Zürich (http://www.gdc.ethz.ch).

The amplicon sequence data preparation followed the workflow established by GDC. Briefly, the raw data were first quality‐controlled using Usearch (v11.0.667) to establish the parameters for the workflow. The reads were cleaned (e.g., PhiX removal and low complexity filter), trimmed from both ends, and read pairs were merged (Usearch v11.0.667, step B). The full‐length primer sequences were then trimmed from the merged reads (Usearch v11.0.667) followed by a filtering step (e.g., mean quality, GC range, size range) using PRINSEQ‐lite (0.20.4). The filtered sequences were clustered with a zero % identity radius (zOTU—Usearch::UNOISE3) with a minimum abundance size of 7. The clustering resulted in 4431 zOTUs. Usearch::SINTAX in combination with SILVA SSU (v128) was used to predict taxonomic associations.

After filtering, a total of 12,354,440 reads with an average of 128,692 reads per sample were obtained (minimum reads = 64,255; maximum reads = 172,467; see Table S1). All four negative controls reached relatively high number of reads, comparable to true animal samples (see Table S1; Figure S2A), which prevented a normalisation and deletion of contaminant OTUs. Therefore, the negative control samples were subjected to more detailed analyses to explore the sources of the potential contamination (see Microbiome analyses for more details). All OTUs which were taxonomically unidentified at the phylum level were removed from the dataset. Likewise, OTUs assigned as “Chloroplast” or “Cyanobacteria” or phyla present only in a single sample were removed. We did not rarefy the dataset to avoid a sampling bias but checked whether all samples reached the plateau phase in a rarefaction curve (Figure S2B).

### Statistical analyses

#### Host growth, survival and feeding rate analyses

We used a proportional hazards Cox regression analysis using water treatment (factor with two levels: W+ and W−), faeces treatment (factor with two levels: F+ and F−), and their interaction as categorical fixed factors. Survival probability over time (i.e., number of hours after exposure to treatment) for four experimental groups was visualised using Kaplan–Meier curves.

For the analysis of growth and food consumption, we used linear mixed models (LMM) again with water treatment (W+ and W−) and faeces treatment (F+ and F−) as fixed factors. Female (brood) ID was included as a random factor and initial body size as a covariate in the models. The Satterthwaite approximation was used to compute degrees of freedom. In addition, we further explored the effect of initial juvenile body size on growth using a simple Pearson correlation.

#### Microbiome analyses

All the microbiome analyses were done separately for each of the four datasets: donors, donor faeces, early recipient juveniles, and recipient juveniles. Phylogenetic information, OTU count data and sample metadata were combined in a single *phyloseq* object. Measures for alpha‐diversity of the bacterial communities were calculated as Shannon diversity, Simpson Evenness, and Chao1 indices. We used linear models to test for the fixed effect of water and faeces treatment as well as their interaction, using sequencing depth (library size) as a covariate. Brood (female) ID was added as a random factor to the dataset of the recipient juveniles. To investigate differences in community composition, we estimated beta‐diversity indexes: Bray–Curtis (relative abundance of OTUs), weighted and unweighted Unifrac (presence/absence of OTUs accounted for their phylogeny and for the relative abundances in weighted Unifrac only), followed by principal coordinates plots (PCoA) based on the matrix distances. The effects of the water and faeces treatments as well as their interaction on the beta‐diversity were assessed using a permutation MANOVA (multivariate analysis of variance; Adonis test). Relative abundance plots were created using the software MicrobiomeAnalyst (Dhariwal et al., 2017) on the level of phylum and genus. Differentially abundant taxa were identified on class and order level among treatments using the *DESeq2* package.

Because the negative control samples showed high sequence counts (Table S1, Figure S2A), we performed additional analyses to explore the sources of potential contamination. Principal coordinate plots show the similarity of bacterial composition among the negative controls as well as the similarity between the controls and the juvenile samples, but not with the donor samples (Figure S3A). This pattern was likely explained by a similar bacterial community composition between negative controls and juvenile samples (Figure S3B,C). The analysis based on differential abundant analysis (DESeq2) showed *Brachybacterium* spp., *Stenotrophomanas* spp. and unidentified *Anaplasmataceae* as the most abundant bacterial members (see Table S2). These bacterial taxa represent environmental microbes typically found in water and soil (Ryan et al., 2009; Tak et al., 2018; Zhang et al., 2007) or obligate intracellular bacteria mainly transmitted by arthropods (family *Anaplasmataceae* including *Wolbachia* spp., Pruneau et al., 2014), and comprised more than 88% of all sequences in control samples (Table S2). Although we have additionally identified contaminating sequences matching water‐ and soil‐associated bacterial genera including *Acinetobacter, Corynebacterium, Micrococcus*, *Propionibacterium*, *Pseudomonas,* and *Streptococcus* (Hammer et al., 2015; Salter et al., 2014), they were represented by negligible sequence counts (see Table S2 for the relative abundances). Although the presence of contaminant DNA is widespread in 16S studies, the effects are particularly problematic in low‐biomass samples that contain very little endogenous DNA (Eisenhofer et al., 2019). This was likely the case in our study since we had to increase the number of PCR cycles due to the poor amplification of juvenile samples (32 instead 31 cycles, see details in Experimental procedures). The observed similarity between the control and true samples suggests that OTUs identified in negative controls may be products of cross‐contamination from true samples, and the removal of such sequences present in negative controls is generally not advised (Hornung et al., 2019) as the OTUs might be part of the core microbiota, with putative physiological relevance for the host (Díaz et al., 2021).

All analyses were performed in R (v. 4.2.2) using packages *phyloseq* (McMurdie & Holmes, 2013), *vegan* (Oksanen et al., 2015), *microbiome* (Lahti & Shetty, 2018) and *DESeq2* (Love et al., 2014) for analyses of microbial diversity and community composition. Packages *survminer* (Kassambara et al., 2020) and *survival* (Therneau, 2023) were used for analyses of survival curves. Packages *lme4* (Bates et al., 2015) and *lmerTest* (Kuznetsova et al., 2014) were used to run linear mixed‐effects models, employing type III Anova and Satterthwaite's approximation for degrees of freedom. We used *ggplot2* (Wickham, 2009) to produce plots. The figures generated in R and MicrobiomeAnalyst were further polished in Inkscape (https://inkscape.org).

## RESULTS

### The effect of experimental treatments on host phenotypic traits

**FIGURE 1:**
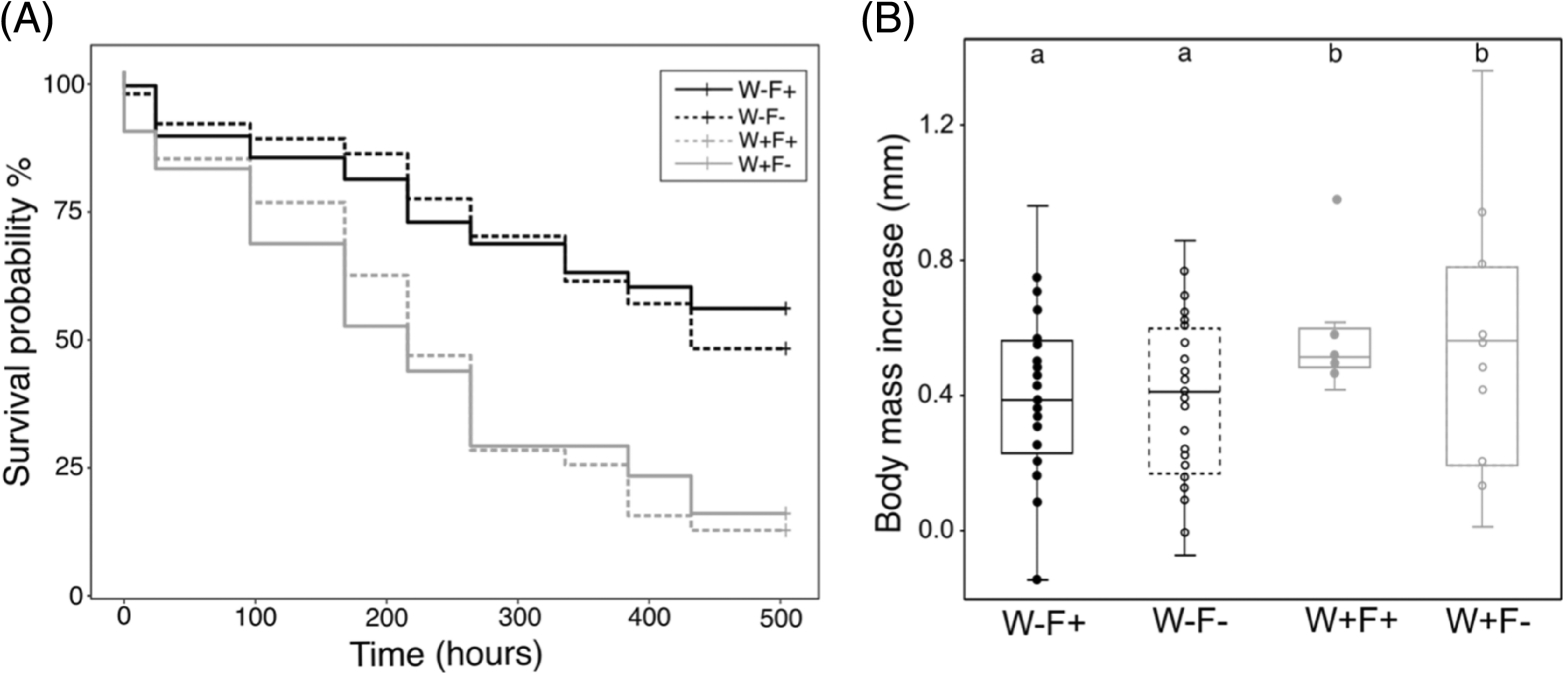
(A) Kaplan–Meier curves for water and faeces experimental treatments of recipient juveniles of a freshwater isopod *Asellus aquaticus*. Juvenile isopods were reared either in polluted (i.e., presence of MPs) or unpolluted water (W+ and W−, respectively) supplemented with the faecal transplant from polluted or unpolluted donor population (F+ and F−; see Figure S1). A significant difference in survival probability was found between the water treatments (Cox regression; *z* = 5.077; *p* < 0.001), with W−F− and W−F+ having the highest survival followed by the W + F+, W + F− group. (B) The effect of micropollutants and faecal microbiome transplant on growth rate of juveniles. Recipient juveniles attained significantly larger final body size in the presence of micropollutants in the water (W+) in comparison to juveniles from W− group (ANOVA: F_*1,87*_ = 7.00, *p* = 0.009). The effect of faeces treatment was not significant (*p* > 0.05).

Mean initial juvenile size did not differ significantly between our four experimental groups W + F+, W + F−, W−F+, W−F− (one‐way ANOVA: *F*_*3,281*_ = 0.276, *p* = 0.843) and was on average: 1.09 mm ± 0.35 SD. There was a significant effect of the water treatment on the survival of juvenile *A. aquaticus* (Cox regression: *z* = 5.077, *p* < 0.0001). Juveniles reared in water with MPs (W+ groups) had over 30% higher chances to die in comparison to juveniles reared in water without any MPs (W− groups) during the experimental period of 21 days (Figure 1A; Hazard ratio = 3.037). There was no statistically significant effect of the faeces treatment (Cox regression: *z* = 0.617, *p* = 0.537) nor of the interaction between water and faeces treatments (Cox regression: *z* = −0.448, *p* = 0.654).

Juvenile growth was significantly affected by the water treatment (Figure 1B; ANOVA: *p* = 0.009; Table S3) and by the initial body size (*p* = 0.025; Table S3). Juveniles showed a higher body mass increase in the presence of MPs (group W+) than juveniles reared in micropollutant‐free water (group W−) (Figure 1B). Initially smaller juveniles showed higher growth, which even translated into a negative correlation between initial and final body sizes (*Pearson: R* ^2^ = −0.07, *p =* 0.009). There was no effect of the faeces treatment on growth (*p* > 0.05; Table S3) nor was there a significant interaction between faeces and water treatments (*p* > 0.05; Table S3). Brood identity did not have a significant effect on growth (*X* ^2^_1_ = 0.096; *p* = 0.757).

Food consumption did not show differences between experimental treatments (ANOVA: all *p* > 0.05; Table S3), nor was it influenced by initial size (*p* > 0.05; Table S3) or brood identity (*X* ^2^_1_ = 0.506; *p* = 0.477).

### The effect of experimental treatments on the host microbiome

#### Donor adults (whole bodies and faecal pellets) and early recipient juveniles

**FIGURE 2:**
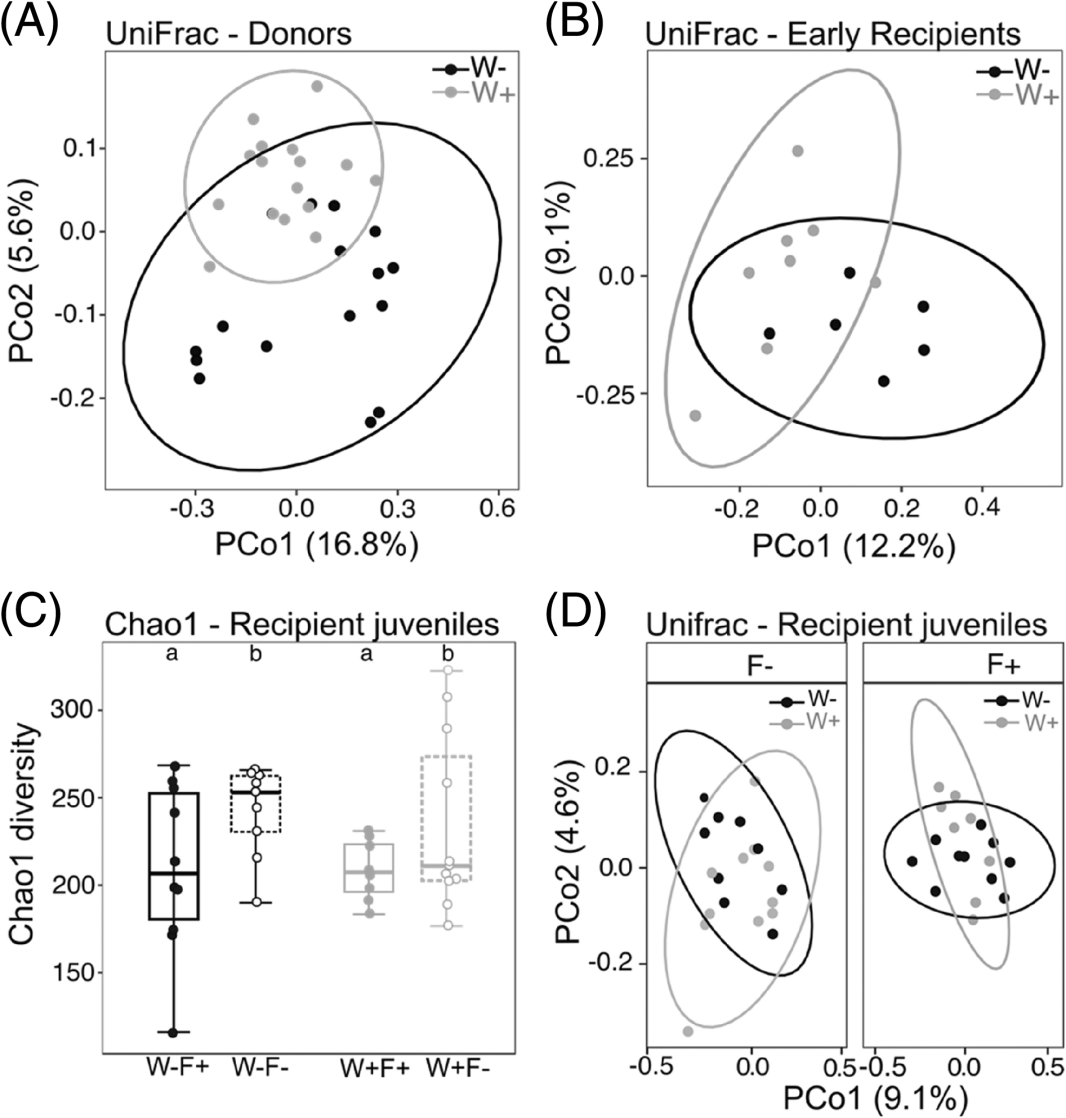
Principal coordinate analysis (PCoA) based on unweighted Unifrac distances showing the degree of similarity between isopod bacterial communities of (A) donor adults and (B) early recipient juveniles (i.e., sacrificed 48 h after the faecal microbiome transplants). Both donor individuals and early recipient juveniles shared more similar bacterial community when exposed to water with MPs (Adonis test Donors: *R* ^2^ = 1.543; *p* = 0.038; Early recipients: *R* ^2^ = 1.184; *p* = 0.034). In early recipient juveniles (B), the faeces treatment did not shape the bacterial community composition (*p* > 0.05; Table S5). In recipient juveniles, (C) bacterial diversity (Chao1 index) is significantly lower in individuals transplanted with a faecal microbiome previously exposed to MPs compared to recipient juveniles with standard microbiome transplants (ANOVA: F_*1,33*_ = 4.554; *p* = 0.040), while the effect of water treatment was not significant (*p* > 0.05; Table S4). (D) The principal coordinate (PCoA) and Adonis analyses based on unweighted Unifrac distances showed a significant interaction between faeces and water treatment on bacterial community composition (Adonis test: *R* ^2^ = 1.199; *p* = 0.050; Table S5). Each data point corresponds to an individual microbiome sample.

We did not find a significant effect of experimental treatments on alpha‐diversity of donor individuals (ANOVA: all indexes *p* > 0.05, see Table S4). However, the MPs induced significant changes in bacterial community composition of donor isopods, with individuals reared in water with MPs showing a more similar microbiome with each other than with individuals reared in a clean water (Figure 2A, unweighted Unifrac; Adonis test: *R* ^2^ = 1.543; *p* = 0.038; Table S5). The dominant taxa were represented by members of *Proteobacteria*, *Bacteroidota*, *Actinomycetota* and *Tenericutes* (Figure S4). In addition, we have identified five differentially abundant OTUs from the orders *Flavobacteriales* and *Deltaproteobacteria* that significantly differed between MP+ and MP− treatments (Figure S5A; DESeq2 test). They were represented by the genera *Flavobacterium*, *Bdellovibrio*, and *Aetherobacter*, respectively (Figures S4B and S5A). The bacterial diversity of donor faeces was significantly lower in the MP+ compared to the MP− treatment (ANOVA: all indexes <0.05, Table S4). The bacterial community composition of faeces was not significantly different between MP treatments (Adonis test: all indexes *p* > 0.05; Table S5), however the analyses of both alpha and beta diversity were performed with very limited sample size (*n* = 3 faecal pellets per MP treatment). The taxonomic composition and relative abundances of community members of faeces was generally similar to the donor isopods, but with higher abundance of *Actinomycetota* and lower abundance of *Bacteroidota* (see Figure S4A–C).

We did not find an effect of water and faecal treatments on alpha‐diversity indexes in early recipient juveniles, which were sacrificed 48 h after the faecal microbiome transplant (ANOVA: all indexes *p* > 0.05, Table S4). The presence of MPs significantly changed their bacterial community composition, and individuals reared in water with MPs for 48h shared more similar microbiomes than when compared to individuals reared in a clean water (Figure 2B, unweighted Unifrac; Adonis test: *R* ^2^ = 1.184; *p* = 0.034; Table S5). The effect of faecal treatment on beta‐diversity (F− *vs.* F+ treatment) was not significant (Adonis test: all indexes *p* > 0.05, Table S5). The taxonomic composition of early recipient juveniles was different in comparison to donor samples and the dominant bacterial phyla were represented by members of *Proteobacteria*, *Actinomycetota*, and *Bacillota* (Figure S4A), with *Brachybacterium* sp. being the most abundant genus among all the samples (Figure S4B). The differences in bacterial community composition between W+ and W− treatment were further explored with DESeq2 test, which showed 16 differentially abundant OTUs from 13 different bacterial orders (Figure S5B), and included bacterial genera such as *Flavobacterium*, *Sphingomonas*, *Legionella*, and *Kocuria* (Figure S5B).

#### Recipient juveniles

**FIGURE 3:**
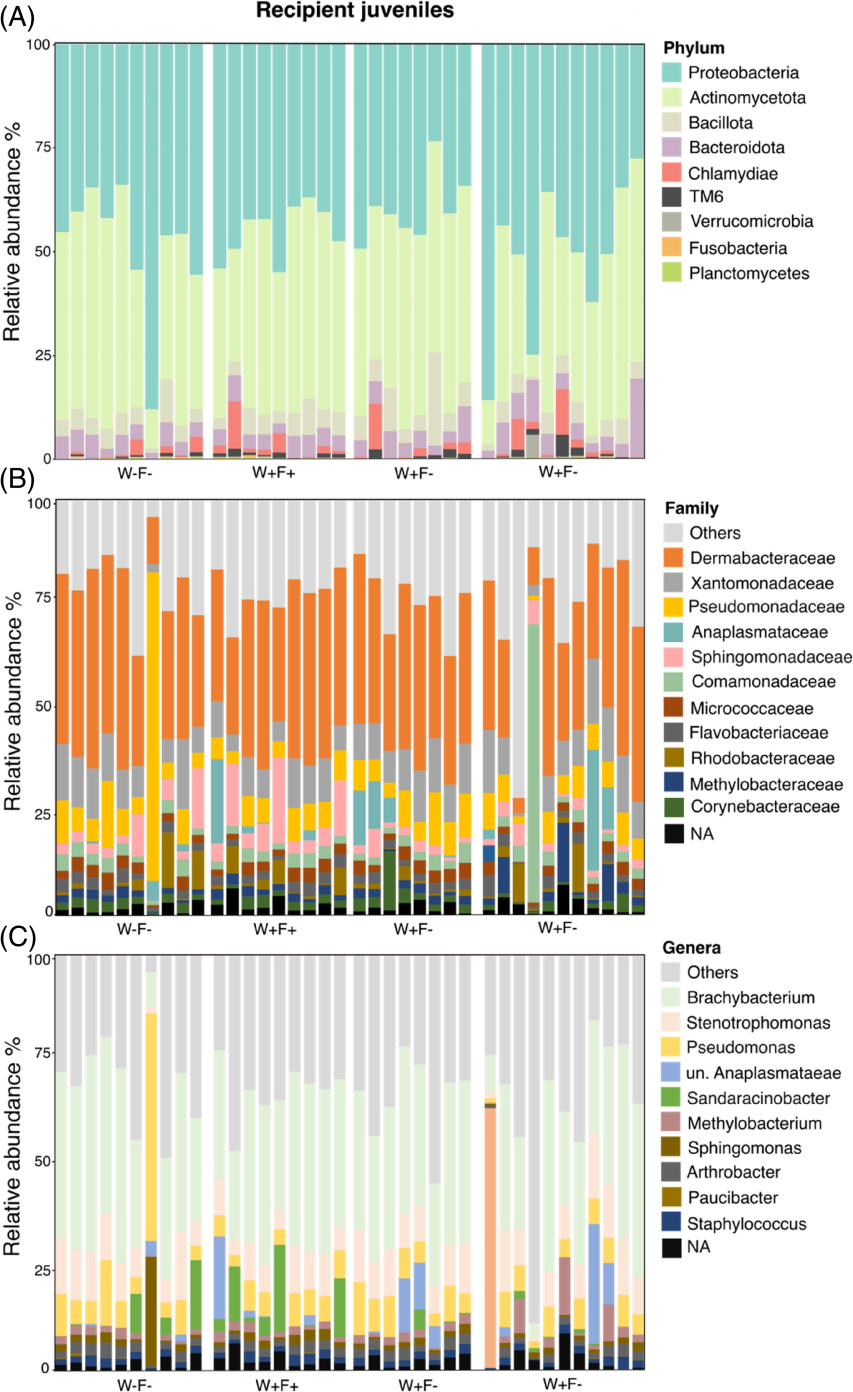
Relative abundance (%) of the bacterial phyla (A) and the most abundant families (B) and genera (C) identified in microbiome of recipient juveniles of a freshwater isopod *Asellus aquaticus*. Other families and genera that were represented by low abundances can be found in Table S6.

Bacterial diversity of recipient juveniles was significantly affected by the faeces treatment (Figure 2C; Chao1 index; ANOVA: faeces treatment; F_*1,33*_ = 4.554; *p* = 0.04; see Table S4) and juveniles transplanted with microbiome exposed to MPs (group F+) showed generally lower bacterial diversity compared to juveniles with standard (F−) transplants (Figure 2C). We also found a significant interaction between faeces and water treatment on bacterial community composition (Figure 2D; unweighted Unifrac; Adonis test: *R* ^2^ = 0.032; *p* = 0.050; Table S5). The presence of MPs in water (group W+) shaped the juvenile microbiome, but only in juveniles transplanted with microbiomes previously exposed to MPs (group W + F+ *vs.* W−F+). There was no clear separation between juveniles with standard (group F−) microbiome transplants when reared in water with or without MPs (group W + F− *vs*. W−F−; Figure 2D; Table S5). In general, the taxonomic composition of recipient juveniles showed a dominance of *Proteobacteria*, *Actinomycetota*, *Bacillota,* and *Bacteroidota* (Figure 3A) with families *Dermabacteraceae*, *Xantomonadaceae*, *Pseudomonadaceae*, and *Anaplasmataceae* (Figure 3B). On the genus level, the most abundant taxa were *Brachybacterium* sp., *Stenotrophomonas* sp., *Pseudomonas* sp., and *Sandaracinobacter* sp. (Figure 3C, Table S6). In addition, we have identified 32 differentially abundant OTUs at the level of bacterial order that differed between experimental treatments (DESeq2 test, Figure S5C), and the differentially abundant OTUs at the level of genus included *Rhizobium* sp., *Legionella* sp., *Flavobacterium* sp. or *Streptomyces* sp., among many others (DESeq2 test, Figure S5C).

## DISCUSSION

Our study showed a strong impact of environmental pollution (i.e., micropollutants) on early survival and growth of the freshwater isopod *A. aquaticus*, an ecologically versatile species considered to be tolerant to a wide range of environmental perturbations (Fraser, 1980; Maltby, 1991; O'Callaghan et al., 2019). We found that exposure to MPs in the water induced changes in the isopod‐associated microbiome. We have successfully isolated and transplanted the faecal microbiomes from donor adults to recipient newly hatched juveniles of *A. aquaticus*, but this transplant did not improve the performance of individuals under MPs exposure. Our results clearly show the negative effects of MPs on a widespread freshwater crustacean, however the involvement of the host microbiome in modulating the toxicity of MPs could not be confirmed.

### The effects of MPs on host performance

The increasing chemical pollution of aquatic ecosystems imposes long‐term adverse effects on aquatic life and ultimately on human health (Landrigan et al., 2020; Malaj et al., 2014; Pinheiro et al., 2021). Micropollutants, which comprise a wide range of low concentration substances including pharmaceuticals, personal care products, industrial chemicals and pesticides (Schwarzenbach et al., 2006), can impact aquatic life at the level of single cells (Ren et al., 2009), individuals (Bundschuh et al., 2012), communities (Munz et al., 2017) or entire ecosystems (Halstead et al., 2014). Our results showed that the exposure to MPs imposed high mortality of juvenile stages of *A*. *aquaticus*, with nearly 50% of individuals not surviving the first weeks of their life. The observed pattern is in agreement with other studies documenting decrease in freshwater species abundance and diversity as well as poor survival of individuals exposed to organic pollution (e.g., Amoatey & Baawain, 2019; Reid et al., 2019; Stamm et al., 2016).

*A. aquaticus* has been described as a species with a great tolerance to environmental pollution, although the mechanisms by which it bioaccumulates and metabolises these toxins are not fully understood (O'Callaghan et al., 2019). There are documented cases with no detectable effects of organic pollution on survival and growth (Bundschuh et al., 2012; Ieromina et al., 2014), while other studies have demonstrated a significant reduction in *A. aquaticus* abundance (Bayona et al., 2015), changes in behaviour (De Lange et al., 2006) or changes in growth rate (Gardeström et al., 2016; Lafuente et al., 2023). The MP mixture of 17 different organic compounds, which mimicked the conditions at impacted sites in Swiss streams (Stamm et al., 2016), has been also applied to other freshwater animals, but it generally showed mixed results. For example, survival of gammarid amphipods exposed to MPs was dependent on their concentration and the life‐stage, with gammarid juveniles showing greater mortality than adults, but only in response to low concentrations of MPs (Taddei et al., 2021). In the freshwater snail *Lymnaea stagnalis*, MP mixture did not have any significant effect on survival, but the study included only adult stages (Salo et al., 2017). Higher sensitivity to organic and heavy metal pollution in early‐life developmental stages has been well documented in *A. aquaticus* (de Nicola et al., 1987; Graça et al., 1994; Naylor et al., 1990), and is consistent with the observed increase in juvenile mortality we observed, even though the adult donor individuals did not show increased mortality in water with MPs.

Crustaceans are generally more susceptible to environmental stresses during and just after moulting (Weis et al., 1992), a process that is more frequent in juvenile stages due to their accelerated growth. Early life stages of aquatic organisms (i.e., fish and invertebrates) are generally more sensitive to toxicants than adults (e.g., Azad, 2013; Hutchinson et al., 1998; Lagadic & Caquet, 1998). Surprisingly, we found that despite the higher mortality, the exposure to MPs promoted the growth rate of surviving individuals. Juveniles attained larger final body size in the presence of MPs. However, this cannot be attributed to increase in their feeding rate, as shown previously (Könemann et al., 2019; Plahuta et al., 2017). Faster growth under organic pollution is often associated with a compensatory or catch‐up growth that occurs after recovering from a period of unfavourable environmental conditions (Hector & Nakagawa, 2012; Hsu et al., 2017). Alternatively, higher growth rate could reflect the direct effects of some specific MPs on isopod physiology or diet quality. One such compound is estradiol (i.e., an endocrine disrupting compound; see Ciślak et al., 2023), which can profoundly alter reproductive physiology of aquatic animals even at trace concentrations (DeFur, 2004; Knigge et al., 2021) and ultimately impacts their sexual development (Vandenbergh et al., 2003), population size (Watts et al., 2002) or individual growth (Plahuta et al., 2017). Another compound potentially changing the quality of food resources is methanol, which was used as a solvent for most of the MPs (see Table 1). Methylotrophic bacteria utilise methanol as their carbon source to fuel their growth (Pfeifenschneider et al., 2017) thus enhancing food quality for isopods that are known to prefer microbially colonised food sources (Bloor, 2011; Ihnen & Zimmer, 2008). We have identified members of *Methylobacteriaceae* (e.g., *Methylobacterium* spp., *Microvirga* spp.) and *Methylophilaceae* (e.g., *Methylophilus*; Table S6), which not only utilise the methanol, but also are able to degrade a wide range of organic MPs (Rodríguez et al., 2018, 2021). Complex interactions among specific compound classes with different modes of action and microbial community can also contribute to observed differences in growth rates (Lafuente et al., 2023; Taddei et al., 2021), but identifying the causal effects would require a further study.

### Effects of MPs on the isopod‐associated microbiome

Freshwater isopods derive the majority of gut microbes from the environment (i.e., transient community) as microbial members present in the water or associated with leaves are typically found also in their digestive tract (Liao et al., 2023). The environmental microbes in our study were exposed to the MPs, providing the potential to adjust to the pollution. Hence, we hypothesised that the microbiome (i.e., transient or already established community) could help to buffer the isopod host against the negative effects of MPs through the ability to degrade and metabolise the organic pollutants (Claus et al., 2016). In general, the bacterial community of both (donor) adults and juveniles was dominated by bacterial phyla *Proteobacteria*, *Actinomycetota*, *Bacillota, Bacteroidota,* and *Tenericutes*, that also dominate in other terrestrial and aquatic isopods (Bredon et al., 2020; Dittmer et al., 2016; Horvathova et al., 2016; Horváthová et al., 2019; Lafuente et al., 2023; Liao et al., 2023; Oliveira et al., 2021). The most abundant taxa were members of *Dermabacteraceae (*Actinomycetota), *Micrococcaceae* (Actinomycetota), *Flavobacteriaceae* (Bacteroidota), *Xanthomonadaceae* (Proteobacteria), *Pseudomonaceae* (Proteobacteria), which have been shown to contribute to the lignocellulose degradation in diverse isopods including *A. aquaticus* (Bredon et al., 2020; Delhoumi et al., 2020). In juveniles, the most abundant bacteria were *Brachybacterium* spp., *Stenotrophomonas* spp., *Pseudomonas* spp., and unidentified species of the *Anaplasmataceae* family. *Brachybacterium*, *Stenotrophomonas and Pseudomonas* spp. are all typical environmental bacteria with some degree of degradation of lignocellulose (Wang et al., 2020; Zhang et al., 2007), xenobiotics (Ryan et al., 2009) or synthetic plastics (Wilkes & Aristilde, 2017). The family *Anaplasmataceae*, also abundant in donor samples, comprises exclusively obligate intracellular bacteria mainly transmitted by arthropods, including *Wolbachia* (Bouchon et al., 1998; Dittmer et al., 2016; Pruneau et al., 2014). As hypothesised, a short exposure to MPs shaped the microbiome in both adult and juvenile stages of *A. aquaticus*. The largest contributors to the observed differences between the control and MP treatment included, among many others, genera *Rhizobium*, *Legionella*, *Flavobacterium*, *Corynebacterium* and *Streptomyces* (Figure S5). These are typical environmental microbes with diverse metabolic repertoires, which were also recently retrieved in a survey of the *A. aquaticus* microbiome originating from the same population (Lafuente et al., 2023). The effect of MPs was observed not only in terms of bacterial community structure, but also bacterial diversity. Juveniles transplanted with a microbiome from MP‐exposed donors showed lower bacterial diversity compared to juveniles with control microbiome transplants. These changes in microbiome in response to MPs can result from different processes. The organic pollutants have generally a strong direct impact on bacterial communities (Rodríguez et al., 2018) and pollutants can shift the community towards taxa that are able to metabolise and utilise the pollutants as a food source (Claus et al., 2016), and lead to the general reduction in bacterial diversity, although the underlying mechanisms are not fully understood (Hernandez et al., 2021; Rocca et al., 2019).

The presence of contaminant DNA is widespread in 16S studies, especially in low‐biomass samples (Eisenhofer et al., 2019). DNA extraction kits, laboratory reagents or researchers themselves are considerable sources of contamination in microbiome studies, and the main steps for minimising the effects of contaminants typically include the use of positive and negative samples, decontaminating working area, sample randomisation, and keeping records of kits and other reagents (Hammer et al., 2015; Salter et al., 2014). Although we followed these general recommendations, the negative samples showed relatively high sequence counts and the bacterial community composition was similar to that of our juvenile samples (but not to our donor samples) (Figure S3), suggesting cross‐contamination from host tissue samples to the negative controls. Distinguishing laboratory or reagent contamination from cross‐contamination with experimental samples is often challenging and requires a careful inspection of the ecological data before removing all the sequences in negative controls or applying other methods such as modifying abundance thresholds or using subtractive filtering (Eisenhofer et al., 2019). Our analysis showed three dominant bacterial taxa found in both juvenile and negative samples: *Brachybacterium* spp., bacteria typically found and isolated from environmental sources (Tak et al., 2018); *Stenotrophomanas* spp., often described as plant‐associated and probiotic bacteria (Ryan et al., 2009), and an unidentified *Anaplasmataceae*, obligate intracellular bacteria of tissues of many arthropods (Pruneau et al., 2014). In such a scenario, the removal of sequences is generally not advised as cross‐contaminated OTUs might be part of the host‐associated microbiome with potential physiological functions (Díaz et al., 2021).

Although MPs had altered the microbiome of the donors, which in turn had influenced the microbiome of the juvenile recipients, faecal microbiome transplants (pre‐adapted to MPs) did not result in improved juvenile survival. As already mentioned, gut microbiota significantly contribute to the utilisation of plant‐based diets (Bredon et al., 2020), and MPs could have shifted the lignocellulose‐degrading community towards other less beneficial taxa. This could explain the negative effects of MPs on juvenile survival mediated through changes in bacterial community structure (and potentially function). Another explanation is that pollutants exert direct effects on the host physiology, and as a consequence disrupt/change the host‐associated microbiome in terms of both bacterial community structure and diversity. Such indirect effects have been well documented in diverse host systems, but most of the evidence comes from correlational studies (Legan et al., 2022; Petrullo et al., 2022; Suzuki, 2017). Alternatively, the faecal microbiome transplants did not alter the juvenile microbiome sufficiently since juveniles were not axenic upon exposure (see Experimental procedures). The initial colonisation of host tissues by microbes can have long‐lasting consequences for the host both in terms of gut microbiome composition and function as well as host physiology (e.g., Debray et al., 2022; Macke et al., 2017). These processes are called priority effects wherein the order and timing of microbial species arrival determine community assembly trajectories (Decaestecker et al., 2024), and such effects could have masked or reduced the impact of faecal microbiome transplants on juvenile performance. However, our analyses were not based on a functional assessment of bacterial taxa, and therefore any functional inference must be taken with caution. Together, our results are consistent with the contribution of diverse environmental bacteria to utilisation of a plant diet in *A. aquaticus* (Bredon et al., 2019, 2020; Zimmer & Bartholmé, 2003) with some degradational potential for xenobiotics. An important component of *A. aquaticus'* diet are also fungi (Graça et al., 1993), which were not analysed in this study. Future studies should therefore focus on the functional analyses of key microbial taxa present in the host, including host‐associated fungi and environmentally‐acquired microbes, as well as their potential to respond to environmental pollution.

## CONCLUSIONS

Understanding the microbiome beyond taxonomic descriptions and towards a more comprehensive (functional) assessment of the complex interplay between microbial symbionts and the host, and of the environmental conditions shaping these relationships, remains a crucial goal, especially in non‐model systems and in natural settings. The faecal microbiome transplants represent one of the powerful tools providing important mechanistic insights into host‐microbial interactions, although it has so far been limited to only few model organisms (Coon et al., 2022; Guo et al., 2020; Secombe et al., 2021). We have successfully transplanted microbiomes from donor adults to recipient juveniles in the non‐model freshwater isopod *Asellus aquaticus*. Faecal microbiome transplants from adults that were pre‐exposed to MPs did not result in improved survival of juvenile recipients, but they induced changes in their microbiome. The contamination of freshwater habitats with MPs is regarded as an alarming environmental issue since MPs can have detrimental effects on aquatic life. Therefore, future studies could use field‐derived microbiome transplants to improve our understanding of the factors shaping the microbiome acquisition, assembly and effects on host physiology of freshwater organisms. Given the high permeability of freshwater animals in general and *Asellus* specifically to their environment, further investigations will require to assess the relative contribution of environmental and endogenous microbes to the observed variation in host performance and/or host microbiome composition.

## AUTHOR CONTRIBUTIONS

**Terézia Horváthová:** Conceptualization (equal); data curation (equal); formal analysis (equal); funding acquisition (equal); investigation (equal); methodology (equal); supervision (equal); visualization (lead); writing – original draft (lead). **Elvira Lafuente:** Conceptualization (equal); data curation (equal); formal analysis (equal); funding acquisition (equal); investigation (equal); methodology (equal); supervision (equal); visualization (equal); writing – review and editing (equal). **Jo‐Anne Bartels:** Formal analysis (supporting); funding acquisition (equal); investigation (equal); writing – review and editing (supporting). **Jesper Wallisch:** Data curation (supporting); investigation (supporting); methodology (equal); writing – review and editing (supporting). **Christoph Vorburger:** Conceptualization (lead); supervision (lead); validation (lead); writing – review and editing (equal).

## CONFLICT OF INTEREST STATEMENT

The authors declare no conflicts of interest.

## Supporting information


**FIGURE S1.** The schematic representation of the experimental design. The donor population was established by placing 15 adult males and 15 adult females, collected previously from the field, in 1.5 L tanks with ad libitum food. Four tanks were either exposed to micropollutants (MP+) or to a clean water without any MPs (MP−) resulting in 120 individuals per MP treatment. After two‐week incubation period, the faecal pellets were carefully collected from the MP+ and MP− tanks, thus generating F+ (polluted) and F− (unpolluted) faecal microbiome transplants. Subset of donor individuals (*n* = 32) and faecal pellets (*n* = 6) were randomly chosen for further microbiome analyses. The recipient population was established by placing gravid females to well plates filled with a clean water and a piece of leaf. The broods (*n* = 300 juveniles) were equally split and randomly distributed to four experimental treatments: W + F+, W + F−, W−F+, W−F−, which corresponded to two water (W+ and W−) and two faeces treatment (F+ and F−). We used the same MPs stock solution (Table 1) as for donor population. The subset of recipient juveniles sacrificed 48 h after the faecal transplant (“early recipient juveniles,” *n* = 16) and juveniles that survived till the end of experiment (“recipient juveniles,” *n* = 40) were collected for the microbiome analyses (see Table S1).
**FIGURE S2.** (A) The sequence sample size (number of reads) of the isopod samples (blue circle, *n* = 94) and the four negative controls (red circle). Negative DNA controls included two extraction and two PCR controls (see also Table S1). (B) Rarefaction curves from the total dataset. Each line corresponds to individual sample of negative controls, donors (group MP+ and MP−) and juveniles (group W−F−, W−F+, W + F−, W + F+; see also Table S1).
**FIGURE S3.** (A) Principal coordinate (PCoA) analysis based on Bray–Curtis distances showing the degree of similarity between negative control samples and isopod samples (donors, donor faeces, early recipient juveniles and recipient juveniles). Principal coordinate (PCoA) and Adonis analysis based on (B) unweighted Unifrac and (C) Bray–Curtis distances showing the degree of similarity between negative control samples (red circles) and the juvenile samples (blue circles). Each data point corresponds to an individual microbiome sample. The percentage of the variation explained by the plotted principal coordinates is indicated on the axes. Based on differential abundant DESeq2 analysis, the dominant bacterial members identified in the negative control samples were also overrepresented in a majority of juvenile samples (see Table S2) and were likely products of cross‐contamination.
**FIGURE S4.** Relative abundance (%) of the bacterial phyla (A) and the most abundant families (B) and genera (C) identified in microbiome of donors, donor faeces and early recipient juveniles of a freshwater isopod *Asellus aquaticus*. Other families and genera that were represented by low abundances can be found in Table S6.
**FIGURE S5.** Differential abundance of operational taxonomic units (zOTUs) between (A) MP+ and MP− treatments of donor individuals; between (B) W+ and W− treatments of early recipient juveniles, and between (C) W + F+ and W−F+ treatments of recipient juveniles. Dot plot shows those zOTUs that were significantly differentially abundant between treatments at the taxonomic level of order and genus (DESeq2; *p* adj <0.001). Differential abundance of operational taxonomic units (zOTUs) DESeq2 analysis was based on the two‐group comparison of significant Adonis test (Figure 2, Table S5).

**TABLE S1.** The isopod and control samples used for the microbiome analysis.

**TABLE S2.** Differential abundance analysis (DESeq2) of operational taxonomic units (zOTUs) between control and recipient juvenile samples.

**TABLE S3.** Results of ANOVA showing the effects of faeces treatment (F+ and F−), water treatment (W+ and W−) and their interaction on isopod juvenile growth (A) and food consumption (B). Initial body size was used as a covariate. The Satterthwaite approximation was used to compute degrees of freedom.
**TABLE S4.** The effect of faeces treatment (F+ and F−) and water treatment (W+ and W−) on isopod bacterial alpha‐diversity. The results are presented for the dataset of donors, donor faeces, early recipient juveniles and recipient juveniles for three different indices: (A) Shannon Diversity, (B) Simpson Evenness and (C) Chao1. The differences in bacterial diversity were analysed with simple ANOVA (donors, early recipient juveniles) or mixed ANOVA (recipient juveniles). The sequence depth was used as a covariate and the mother ID as a random factor (only for mixed ANOVA).
**TABLE S5.** The effect of faeces treatment (F+ and F−) and water treatment (W+ and W−) on isopod bacterial beta‐diversity. The results are presented for the dataset of donors, donor faeces, early recipient juveniles and recipient juveniles for three different metrics: (A) Bray–Curtis, (B) unweighted UniFrac and (C) weighted UniFrac distances. The analysis of similarity (measure of beta‐diversity) was based on permutational multivariate analysis of variance (adonis function) with 9999 permutations. The sequence depth (number of sequences per each sample) was used as a covariate.

**TABLE S6.** Relative abundance (%) of the less abundant bacterial families and genera identified in microbiome of recipient juveniles of a freshwater isopod *Asellus aquaticus* (see Figure 3 for the most abundant bacterial members).

## Data Availability

All raw reads can be accessed via the European Nucleotide Archive (ENA) at EMBL‐EBI: Accession Number PRJEB66514.
