# Supplementary material for: Tolerance to environmental pollution in the freshwater crustacean Asellus aquaticus: A role for the microbiome
Source: Environ Microbiol Rep. 2024 May 23;16(3):e13252. doi: 10.1111/1758-2229.13252 (PMC11116767; doi:10.1111/1758-2229.13252)
Supplement: Supplementary file 1 — FIGURE S1. The schematic representation of the experimental design. The donor population was established by placing 15 adult males and 15 adult females, collected previously from the field, in 1.5 L tanks with ad libitum food. Four tanks were either exposed to micropollutants (MP+) or to a clean water without any MPs (MP−) resulting in 120 individuals per MP treatment. After two‐week incubation period, the faecal pellets were carefully collected from the MP+ and MP− tanks, thus generating F+ (polluted) and F− (unpolluted) faecal microbiome transplants. Subset of donor individuals (n = 32) and faecal pellets (n = 6) were randomly chosen for further microbiome analyses. The recipient population was established by placing gravid females to well plates filled with a clean water and a piece of leaf. The broods (n = 300 juveniles) were equally split and randomly distributed to four experimental treatments: W + F+, W + F−, W−F+, W−F−, which corresponded to two water (W+ and W−) and two faeces treatment (F+ and F−). We used the same MPs stock solution (Table 1) as for donor population. The subset of recipient juveniles sacrificed 48 h after the faecal transplant (“early recipient juveniles,” n = 16) and juveniles that survived till the end of experiment (“recipient juveniles,” n = 40) were collected for the microbiome analyses (see Table S1). FIGURE S2. (A) The sequence sample size (number of reads) of the isopod samples (blue circle, n = 94) and the four negative controls (red circle). Negative DNA controls included two extraction and two PCR controls (see also Table S1). (B) Rarefaction curves from the total dataset. Each line corresponds to individual sample of negative controls, donors (group MP+ and MP−) and juveniles (group W−F−, W−F+, W + F−, W + F+; see also Table S1). FIGURE S3. (A) Principal coordinate (PCoA) analysis based on Bray–Curtis distances showing the degree of similarity between negative control samples and isopod samples (donors, donor faeces, early rec [file EMI4-16-e13252-s003.docx]

**
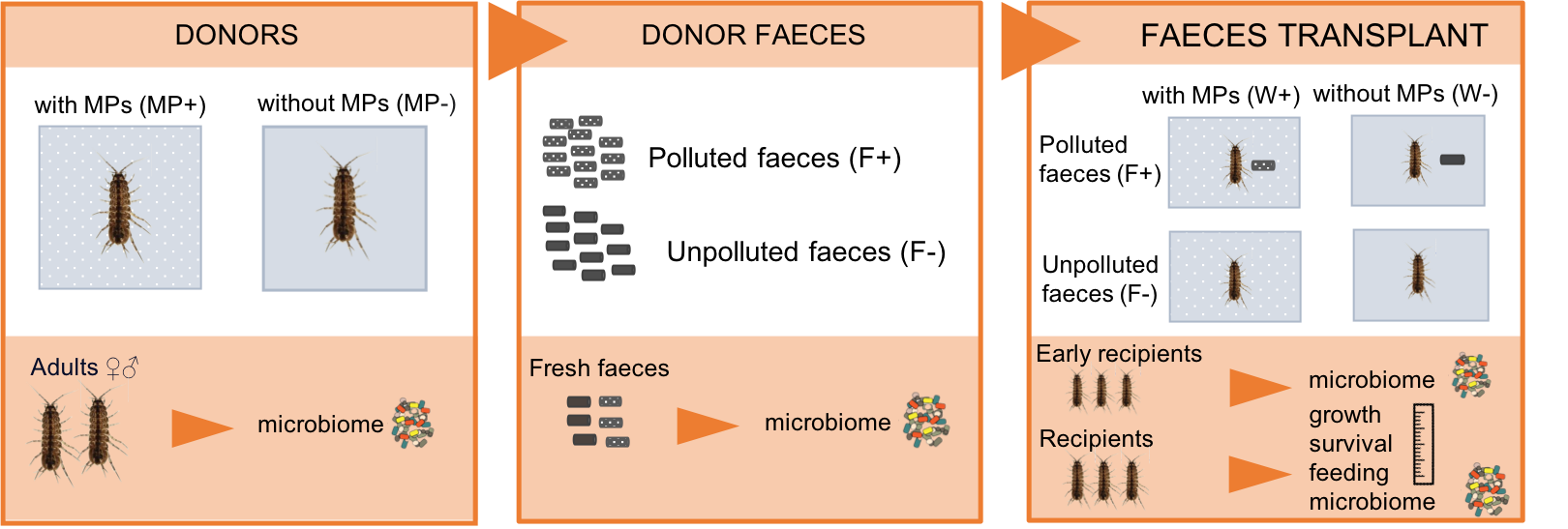
**

**Fig. S1** The schematic representation of the experimental design. The donor population was established by placing 15 adult males and 15 adult females, collected previously from the field, in 1.5 L tanks with *ad libitum* food. Four tanks were either exposed to micropollutants (MP+) or to a clean water without any MPs (MP-) resulting in 120 individuals per MP treatment. After two-week incubation period, the faecal pellets were carefully collected from the MP+ and MP- tanks, thus generating F+ (polluted) and F- (unpolluted) faecal microbiome transplants. Subset of donor individuals (n = 32) and faecal pellets (n = 6) were randomly chosen for further microbiome analyses. The recipient population was established by placing gravid females to well plates filled with a clean water and a piece of leaf. The broods (n = 300 juveniles) were equally split and randomly distributed to four experimental treatments: W+F+, W+F-, W-F+, W-F-, which corresponded to two water (W+ and W-) and two faeces treatment (F+ and F-). We used the same MPs stock solution (Table 1) as for donor population. The subset of recipient juveniles sacrificed 48 hours after the faecal transplant (“early recipient juveniles”, n = 16) and juveniles that survived till the end of experiment (“recipient juveniles”, n = 40) were collected for the microbiome analyses (see Table S1).


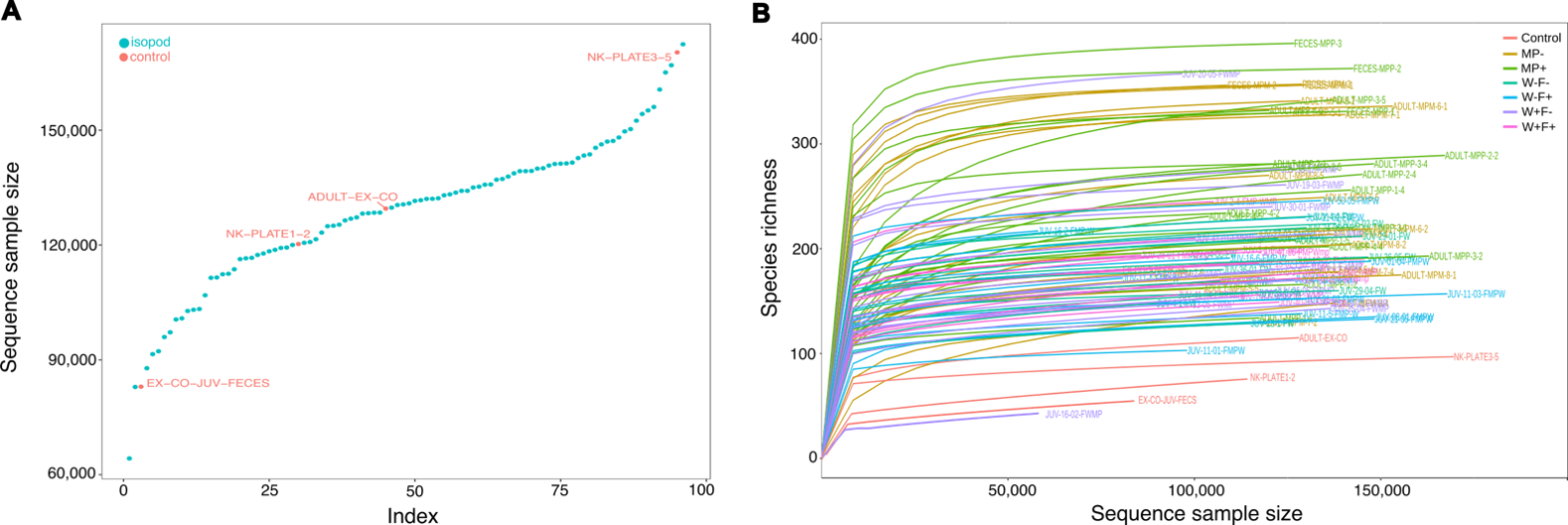


**Figure S2. A)** The sequence sample size (number of reads) of the isopod samples (blue circle, n = 94) and the four negative controls (red circle). Negative DNA controls included two extraction and two PCR controls (see also Table S1). **B)** Rarefaction curves from the total dataset. Each line corresponds to individual sample of negative controls, donors (group MP+ and MP-) and juveniles (group W-F-, W-F+, W+F-, W+F+; see also Table S1).


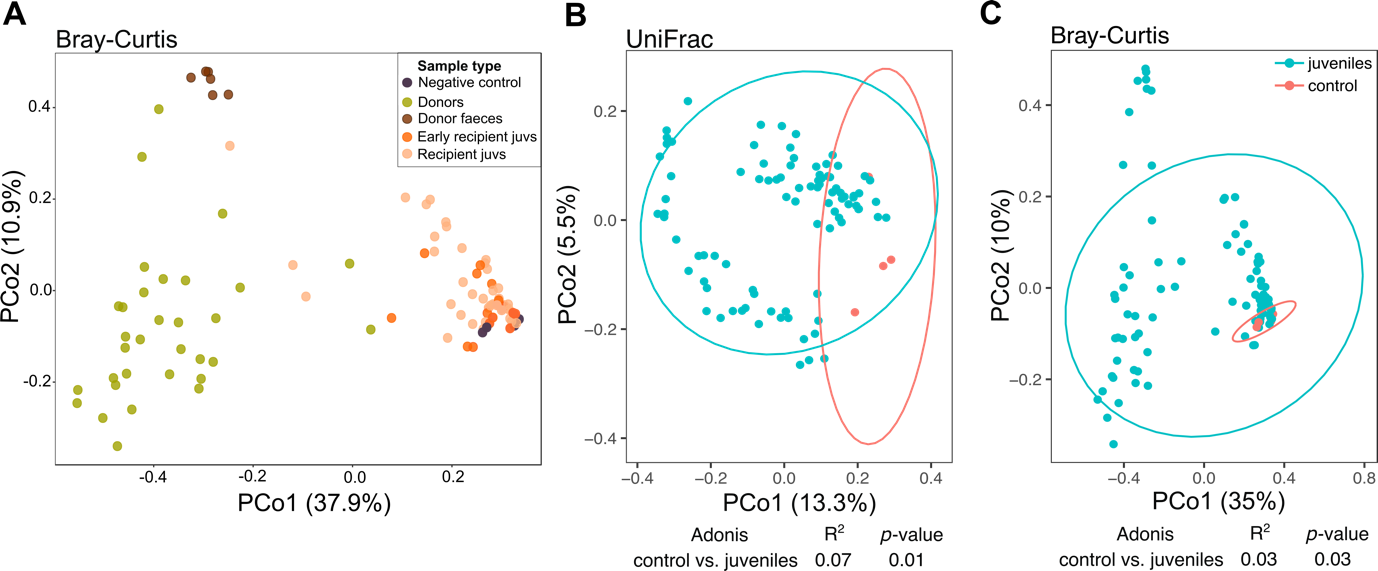


**Figure S3. A)** Principal coordinate (PCoA) analysis based on Bray-Curtis distances showing the degree of similarity between negative control samples and isopod samples (donors, donor faeces, early recipient juveniles and recipient juveniles). Principal coordinate (PCoA) and Adonis analysis based on **(B)** unweighted Unifrac and **(C)** Bray-Curtis distances showing the degree of similarity between negative control samples (red circles) and the juvenile samples (blue circles). Each data point corresponds to an individual microbiome sample. The percentage of the variation explained by the plotted principal coordinates is indicated on the axes. Based on differential abundant DESeq2 analysis, the dominant bacterial members identified in the negative control samples were also overrepresented in a majority of juvenile samples (see Table S2) and were likely products of cross-contamination.


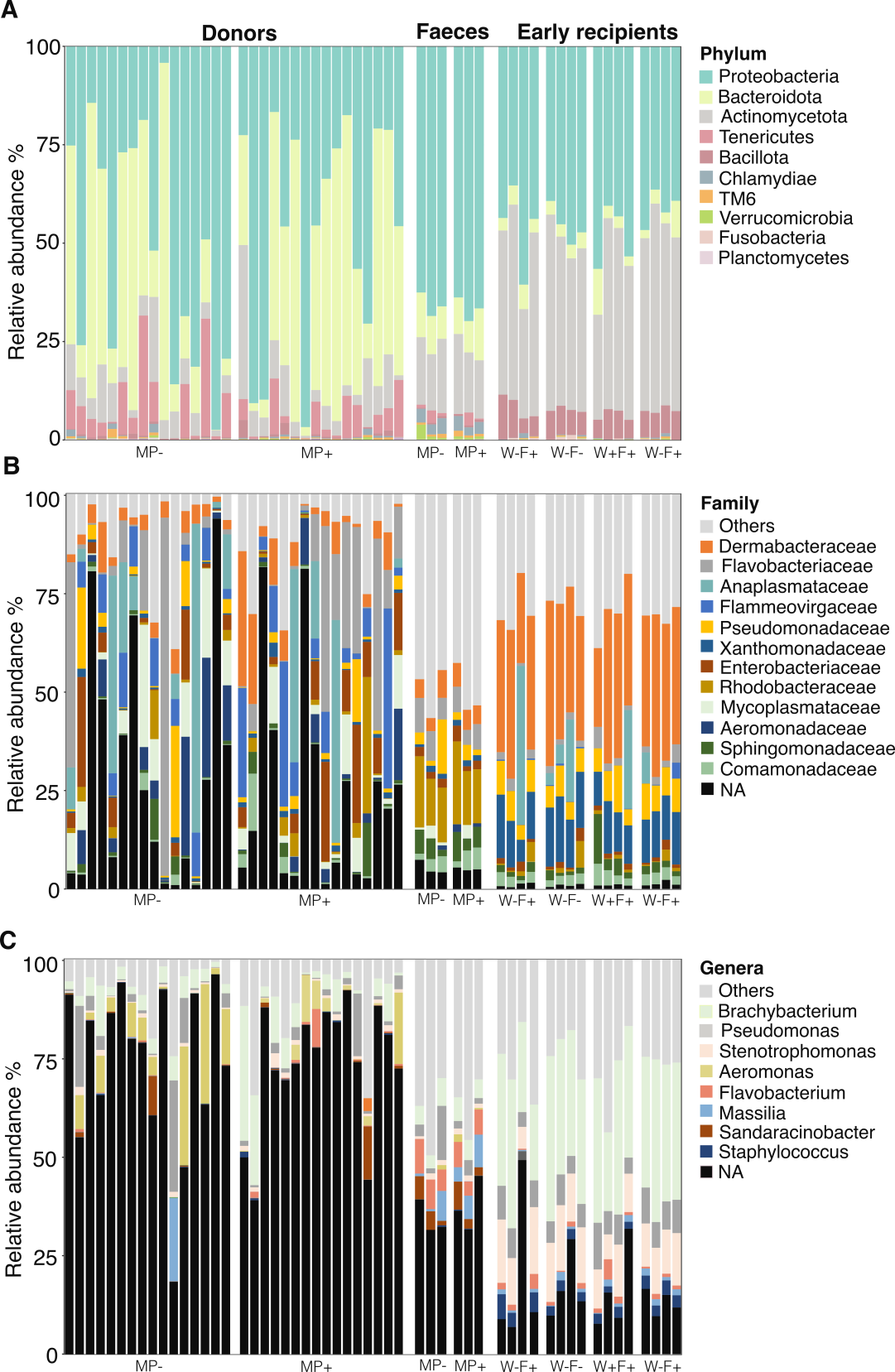


**Figure S4**. Relative abundance (%) of the bacterial phyla **(A)** and the most abundant families (B) and genera **(C)** identified in microbiome of donors, donor faeces and early recipient juveniles of a freshwater isopod *Asellus aquaticus*. Other families and genera that were represented by low abundances can be found in Table S6.


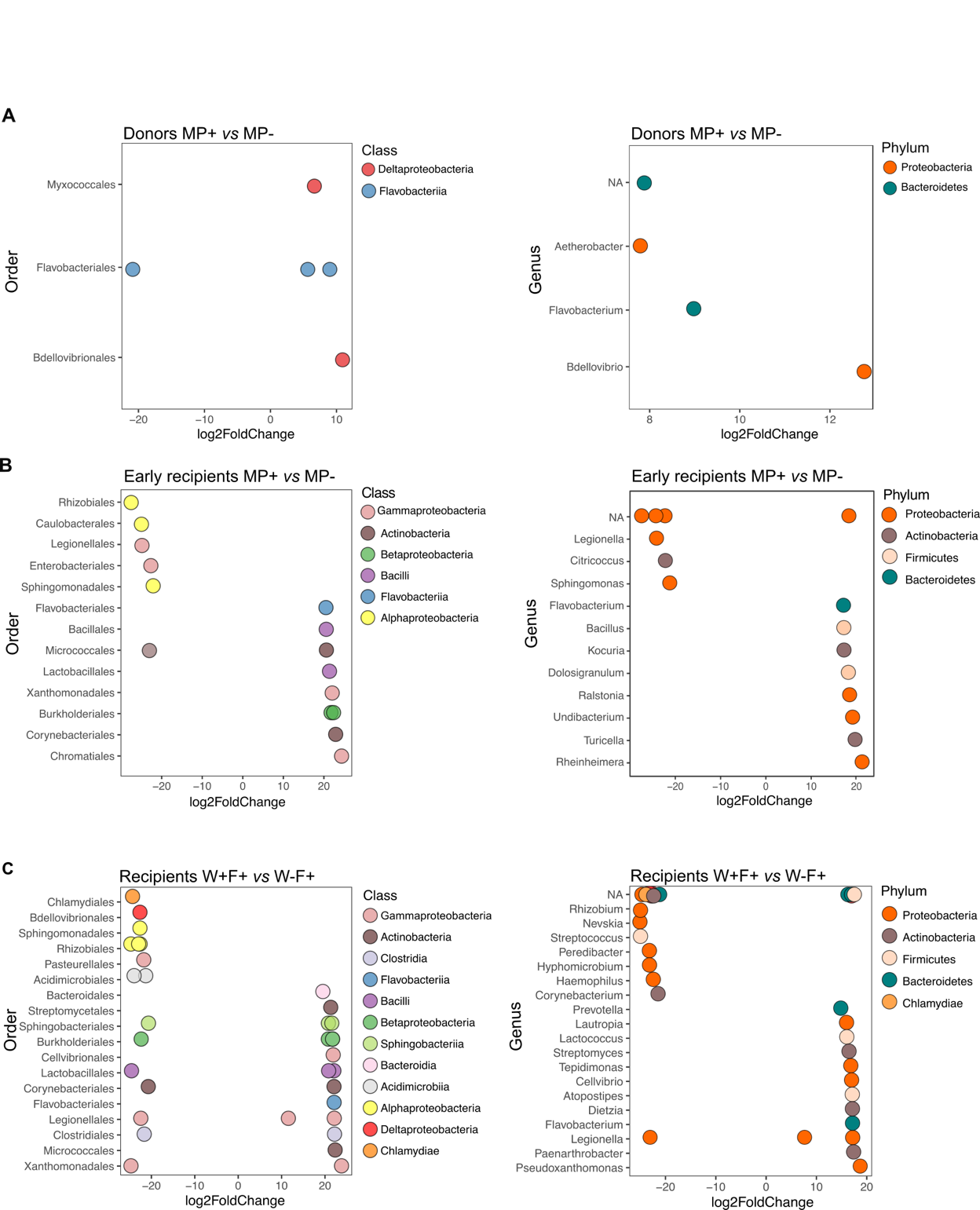


**Figure S5.** Differential abundance of operational taxonomic units (zOTUs) between **(A)** MP+ and MP-treatments of donor individuals; between **(B)** W+ and W-treatments of early recipient juveniles, and between **(C)** W+F+ and W-F+ treatments of recipient juveniles. Dot plot shows those zOTUs that were significantly differentially abundant between treatments at the taxonomic level of order and genus (DESeq2; *p* adj <0.001). Differential abundance of operational taxonomic units (zOTUs) DESeq2 analysis was based on the two-group comparison of significant Adonis test (Fig. 2, Table S5).
