# Supplementary material for: Tolerance to environmental pollution in the freshwater crustacean Asellus aquaticus: A role for the microbiome
Source: Environ Microbiol Rep. 2024 May 23;16(3):e13252. doi: 10.1111/1758-2229.13252 (PMC11116767; doi:10.1111/1758-2229.13252)
Supplement: Supplementary file 4 — TABLE S3. Results of ANOVA showing the effects of faeces treatment (F+ and F−), water treatment (W+ and W−) and their interaction on isopod juvenile growth (A) and food consumption (B). Initial body size was used as a covariate. The Satterthwaite approximation was used to compute degrees of freedom. TABLE S4. The effect of faeces treatment (F+ and F−) and water treatment (W+ and W−) on isopod bacterial alpha‐diversity. The results are presented for the dataset of donors, donor faeces, early recipient juveniles and recipient juveniles for three different indices: (A) Shannon Diversity, (B) Simpson Evenness and (C) Chao1. The differences in bacterial diversity were analysed with simple ANOVA (donors, early recipient juveniles) or mixed ANOVA (recipient juveniles). The sequence depth was used as a covariate and the mother ID as a random factor (only for mixed ANOVA). TABLE S5. The effect of faeces treatment (F+ and F−) and water treatment (W+ and W−) on isopod bacterial beta‐diversity. The results are presented for the dataset of donors, donor faeces, early recipient juveniles and recipient juveniles for three different metrics: (A) Bray–Curtis, (B) unweighted UniFrac and (C) weighted UniFrac distances. The analysis of similarity (measure of beta‐diversity) was based on permutational multivariate analysis of variance (adonis function) with 9999 permutations. The sequence depth (number of sequences per each sample) was used as a covariate. [file EMI4-16-e13252-s004.docx]

**Table S3.** Results of ANOVA showing the effects of faeces treatment (F+ and F-), water treatment (W+ and W-) and their interaction on isopod juvenile growth **(A**) and food consumption **(B)**. Initial body size was used as a covariate. The Satterthwaite approximation was used to compute degrees of freedom.

|  | **NumDF (DenDF)** | **SumOfSqs** | **MeanSq** | **F** | **Pr(>F)** |
| --- | --- | --- | --- | --- | --- |
| **A) Growth** |  |  |  |  |  |
| Faeces | 1 (82) | 0.003 | 0.003 | 0.054 | 0.817 |
| Water | 1 (88) | 0.397 | 0.397 | 7.001 | **0.009** |
| Faeces x water | 1 (81) | 0.000 | 0.000 | 0.003 | 0.056 |
| Initial size | **1 (18)** | 0.342 | 0.342 | 6.026 | **0.025** |
| **B) Food consumption** |  |  |  |  |  |
| Faeces | 1 (82) | 14.064 | 14.064 | 0.001 | 0.982 |
| Water | 1 (87) | 1.078 | 1.078 | 0.541 | 0.464 |
| Faeces x water | 1 (81) | 30.513 | 30.513 | 0.041 | 0.839 |
| Initial size | 1 (25) | 0.014 | 0.014 | 1.173 | 0.282 |

**Table S4.** The effect of faeces treatment (F+ and F-) and water treatment (W+ and W-) on isopod bacterial alpha-diversity. The results are presented for the dataset of donors, donor faeces, early recipient juveniles and recipient juveniles for three different indices: **A)** Shannon Diversity, **B)** Simpson Evenness and **C)** Chao1. The differences in bacterial diversity were analysed with simple ANOVA (donors, early recipient juveniles) or mixed ANOVA (recipient juveniles). The sequence depth was used as a covariate and the mother ID as a random factor (only for mixed ANOVA).

|  | **Df** | **SumOfSqs** | | **MeanSq** | **F** | **Pr(>F)** |
| --- | --- | --- | --- | --- | --- | --- |
| **A) Shannon-Diversity** |  |  | |  |  |  |
| **Donors** |  |  | |  |  |  |
| Sequence depth | 1 | 1.891 | | 1.890 | 2.989 | 0.094 |
| Water | 2 | 0.138 | | 0.069 | 0.109 | 0.897 |
| Residuals | 32 | 20.242 | | 0.633 |  |  |
| **Donor faeces** |  |  | |  |  |  |
| Sequence depth | 1 | 0.1025 | | 0.103 | 0.583 | 0.474 |
| Water | 2 | 11.371 | | 5.685 | 32.319 | **0.001** |
| Residuals | 6 | 1.056 | | 0.176 |  |  |
| **Early recipient juveniles** |  |  | |  |  |  |
| Sequence depth | 1 | 0.006 | | 0.006 | 0.031 | 0.865 |
| Water | 1 | 0.095 | | 0.095 | 0.495 | 0.500 |
| Faeces | 1 | 0.061 | | 0.061 | 0.319 | 0.586 |
| Water:Faeces | 1 | 0.056 | | 0.056 | 0.292 | 0.602 |
| Residuals | 9 | 1.733 | | 0.193 |  |  |
| **B) Simpson Evenness** |  |  | |  |  |  |
| **Donors** |  |  | |  |  |  |
| Sequence depth | 1 | 0.001 | | 0.001 | 4.167 | **0.050** |
| Water | 2 | 0.000 | | 0.000 | 0.267 | 0.767 |
| Residual | 32 | 0.005 | | 0.000 |  |  |
| **Donor faeces** |  |  | |  |  |  |
| Sequence depth | 1 | 0.001 | | 0.001 | 4.579 | 0.076 |
| Water | 2 | 0.005 | | 0.002 | 8.737 | **0.017** |
| Residuals | 6 | 0.002 | | 0 |  |  |
| **Early recipient juveniles** |  |  | |  |  |  |
| Sequence depth | 1 | 0 | | 0 | 1.455 | 0.259 |
| Water | 1 | 0 | | 0 | 0.002 | 0.966 |
| Faeces | 1 | 0 | | 0 | 0.516 | 0.491 |
| Water:Faeces | 1 | 0 | | 0 | 0.000 | 0.995 |
| Residual | 9 | 0.001 | | 0 |  |  |
| **C) Chao1** |  |  | |  |  |  |
| **Donors** |  |  | |  |  |  |
| Sequence depth | 1 | 7649 | | 7648 | 0.857 | 0.361 |
| Water | 2 | 54979 | | 27489 | 3.081 | 0.060 |
| Residuals | 32 | 285562 | | 0.927 |  |  |
| **Donor faeces** |  |  | |  |  |  |
| Sequence depth | 1 | 2 | | 2 | 0.001 | 0.980 |
| Water | 2 | 309542 | | 154771 | 61.502 | **< 0.001** |
| Residuals | 6 | 15099 | | 2517 |  |  |
| **Early recipient juveniles** |  |  | |  |  |  |
| Sequence depth | 1 | 7962 | | 7962 | 2.654 | 0.138 |
| Water | 1 | 13136 | | 13136 | 4.378 | 0.066 |
| Faeces | 1 | 3032 | | 3032 | 1.011 | 0.341 |
| Water:Faeces | 1 | 1437 | | 1437 | 0.479 | 0.506 |
| Residuals | 9 | 27004 | | 3000 |  |  |
|  |  |  | |  |  |  |
|  | **NumDF** | | **DenDF** | **MeanSq** | **F** | **Pr(>F)** |
| **Recipient juveniles** |  |  | |  |  |  |
| **A) Shannon Diversity** |  |  | |  |  |  |
| Sequence depth | 1 | 30.010 | | 0.025 | 0.107 | 0.746 |
| Water | 1 | 32.910 | | 0.003 | 0.012 | 0.912 |
| Faeces | 1 | 31.423 | | 0.211 | 0.894 | 0.352 |
| Water:Faeces | 1 | 29.999 | | 0.359 | 1.521 | 0.227 |
| **A) Simpson Evenness** |  |  | |  |  |  |
| Sequence depth | 1 | 33.000 | | 0 | 0.042 | 0.839 |
| Water | 1 | 33.000 | | 0 | 0.464 | 0.501 |
| Faeces | 1 | 33.000 | | 0 | 0.840 | 0.366 |
| Water:Faeces | 1 | 33.000 | | 0 | 0.088 | 0.769 |
| **C) Chao1** |  |  | |  |  |  |
| Sequence depth | 1 | 33.000 | | 2906 | 0.485 | 0.491 |
| Water | 1 | 33.000 | | 1304 | 0.217 | 0.644 |
| Faeces | 1 | 33.000 | | 27315 | 4.554 | **0.040** |
| Water:Faeces | 1 | 33.000 | | 257 | 1.110 | 0.330 |

**Table S5.** The effect of faeces treatment (F+ and F-) and water treatment (W+ and W-) on isopod bacterial beta-diversity. The results are presented for the dataset of donors, donor faeces, early recipient juveniles and recipient juveniles for three different metrics: **A)** Bray-Curtis, **B**) unweighted UniFrac, and **C)** weighted UniFrac distances. The analysis of similarity (measure of beta-diversity) was based on permutational multivariate analysis of variance (adonis function) with 9999 permutations. The sequence depth (number of sequences per each sample) was used as a covariate.

|  | **Df** | **SumOfSqs** | **R^2^** | **F** | **Pr(>F)** |
| --- | --- | --- | --- | --- | --- |
| **A) Bray-Curtis** |  |  |  |  |  |
| **Donors** |  |  |  |  |  |
| Sequence depth | 1 | 0.673 | 0.068 | 2.215 | **0.008** |
| Water | 1 | 0.361 | 0.037 | 1.164 | 0.307 |
| Residual | 29 | 8.812 | 0.895 |  |  |
| **Donor faeces** |  |  |  |  |  |
| Sequence depth | 1 | 0.095 | 0.214 | 1.165 | 0.271 |
| Water | 1 | 0.104 | 0.234 | 1.273 | 0.204 |
| Residual | 3 | 0.245 | 0.552 |  |  |
| **Early recipient juveniles** |  |  |  |  |  |
| Sequence depth | 1 | 0.035 | 0.047 | 0.579 | 0.893 |
| Water | 1 | 0.065 | 0.089 | 1.085 | 0.358 |
| Faeces | 1 | 0.047 | 0.065 | 0.791 | 0.760 |
| Water:Faeces | 1 | 0.047 | 0.064 | 0.786 | 0.691 |
| Residual | 9 | 0.540 | 0.735 |  |  |
| **Recipient juveniles** |  |  |  |  |  |
| Sequence depth | 1 | 0.109 | 0.023 | 0.847 | 0.583 |
| Water | 1 | 0.052 | 0.022 | 0.818 | 0.561 |
| Faeces | 1 | 0.073 | 0.031 | 1.139 | 0.278 |
| Water:Faeces | 1 | 0.071 | 0.030 | 1.110 | 0.330 |
| Residual | 33 | 2.114 | 0.896 |  |  |
| **B) Unweighted UniFrac** |  |  |  |  |  |
| **Donors** |  |  |  |  |  |
| Sequence depth | 1 | 0.261 | 0.043 | 1.378 | 0.063 |
| Water | 1 | 0.292 | 0.048 | 1.543 | **0.038** |
| Residual | 29 | 5.495 | 0.909 |  |  |
| **Donor faeces** |  |  |  |  |  |
| Sequence depth | 1 | 0.110 | 0.189 | 0.941 | 0.701 |
| Water | 1 | 0.121 | 0.208 | 1.035 | 0.369 |
| Residual | 3 | 0.350 | 0.603 |  |  |
| **Early recipient juveniles** |  |  |  |  |  |
| Sequence depth | 1 | 0.237 | 0.079 | 1.048 | 0.277 |
| Water | 1 | 0.267 | 0.089 | 1.184 | **0.034** |
| Faeces | 1 | 0.218 | 0.073 | 0.965 | 0.641 |
| Water:Faeces | 1 | 0.243 | 0.081 | 1.078 | 0.188 |
| Residual | 9 | 2.033 | 0.678 |  |  |
| **Recipient juveniles** |  |  |  |  |  |
| Sequence depth | 1 | 0.226 | 0.026 | 0.971 | 0.560 |
| Water | 1 | 0.261 | 0.030 | 1.121 | 0.112 |
| Faeces | 1 | 0.259 | 0.030 | 1.112 | 0.142 |
| Water:Faeces | 1 | 0.279 | 0.032 | 1.199 | **0.050** |
| Residual | 33 | 7.690 | 0.882 |  |  |
| **C) Weighted UniFrac** |  |  |  |  |  |
| **Donors** |  |  |  |  |  |
| Sequence depth | 1 | 0.216 | 0.039 | 1.234 | 0.263 |
| Water | 1 | 0.184 | 0.034 | 1.053 | 0.336 |
| Residual | 29 | 5.073 | 0.927 |  |  |
| **Donor faeces** |  |  |  |  |  |
| Sequence depth | 1 | 0.036 | 0.227 | 1.183 | 0.279 |
| Water | 1 | 0.032 | 0.199 | 1.040 | 0.442 |
| Residual | 3 | 0.091 | 0.574 |  |  |
| **Early recipient juveniles** |  |  |  |  |  |
| Sequence depth | 1 | 0.013 | 0.045 | 0.538 | 0.835 |
| Water | 1 | 0.022 | 0.075 | 0.890 | 0.533 |
| Faeces | 1 | 0.016 | 0.054 | 0.641 | 0.819 |
| Water:Faeces | 1 | 0.021 | 0.073 | 0.869 | 0.524 |
| Residual | 9 | 0.220 | 0.754 |  |  |
| **Recipient juveniles** |  |  |  |  |  |
| Sequence depth | 1 | 0.050 | 0.021 | 0.780 | 0.589 |
| Water | 1 | 0.052 | 0.022 | 0.818 | 0.561 |
| Faeces | 1 | 0.073 | 0.031 | 1.139 | 0.278 |
| Water:Faeces | 1 | 0.071 | 0.030 | 1.110 | 0.330 |
| Residual | 33 | 2.114 | 0.896 |  |  |
